# Evaluation of antidepressant activity of tramadol in mice

**DOI:** 10.4103/0253-7613.42307

**Published:** 2008-06

**Authors:** Vandana Tayal, Bhupinder Singh Kalra, Shalini Chawla

**Affiliations:** Department of Pharmacology, Maulana Azad Medical College, Bahadur Shah Zafar Marg, New Delhi 110 002, India; 1 ITS Dental College, Delhi Meerut Highway, Ghaziabad, UP- 201 206, India

**Keywords:** Antidepressant, tail suspension test, tramadol

## Abstract

**Objective:**

To evaluate antidepressant like effect of tramadol in mice.

**Materials and Methods:**

Tramadol was administered at three different doses (10,20 and 40 mg/kg, i.p) once daily for 7 days to *Swiss* albino mice of either sex. The immobility period of control and drug treated mice were recorded in tail suspension test (TST).The antidepressant effect of tramadol was compared to that of fluoxetine (20 mg/kg, i.p), administered for seven days.

**Results:**

Tramadol produced significant antidepressant effect at all the doses, as indicated by reduction in immobility times as compared to control. The efficacy of tramadol at doses of 20 and 40 mg/kg was comparable with that of fluoxetine. Tramadol at 10 mg/kg dose showed significantly less antidepressant activity compared to fluoxetine.

**Conclusion:**

The results of the present study indicate antidepressant like activity of tramadol.

## Introduction

Tramadol is a synthetic centrally acting opioid analgesic used mainly for the treatment of moderate to severe pain.[1] It is a weak µ opioid receptor agonist and also produces analgesia by inhibiting uptake of norepinephrine and serotonin.[1] Tramadol causes activation of both the systems involved in inhibition of pain i.e. the opioid and the descending monoaminergic pain modulating pathway. There is a large body of evidence to suggest that the analgesic action of tramadol is mainly related to central monoaminergic mechanism rather than opioid receptor pathway. It has also been observed that tramadol induced analgesia is blocked by α_2_ adrenergic receptor antagonist Yohimbine.[2]

*In vitro* studies have shown that tramadol effectively inhibits reuptake of monoamines.[3] It has also been established that tramadol inhibits reuptake of serotonin in the raphe nucleus.[4] Antidepressants mainly act by inhibiting norepinephrine-serotonin reuptake and tramadol by virtue of its property of blocking monoaminergic reuptake may also act as an antidepressant. Additionally, tramadol bears a close structural similarity to antidepressant venlafaxine and thus shares a number of its molecular and pharmacological features.[5] In a study conducted in mice using an experimental model, it was seen that tramadol exhibits antidepressant activity.[1] Another study in rats showed that tramadol led to decreased number of failures to avoid or escape aversive stimuli (shock) in learned helplessness model.[6] Few documented clinical reports have also indicated the possibility of antidepressant effect of tramadol. In one case report, a case of severe suicidal ideation rapidly resolved with intramuscular tramadol.[7] Tramadol monotherapy was also reported to be effective in a case of refractory major depression.[8]

Hence, this study was undertaken with the objective of evaluating the antidepressant like activity of tramadol in an animal model of depression.

## Materials and Methods

This study was conducted after getting an approval by the Institutional Review Board and Animal Ethical Committee. Animals were procured from central animal house and kept in air conditioned environment. After procurement a study gap, of one week was allowed for acclimatization. *Swiss* albino mice of either sex, weighing 20-25gm (age 3 months were used). They were provided with normal diet with water ad libitum.

Animals were divided into 5 groups of 8 mice each. Group 1 (control) was given normal saline (0.1ml/10gm). Group 2, 3 and 4 were treated with 3 different doses (10, 20 and 40mg/kg) of tramadol for 7 days.[9] Group 5 was administered with fluoxetine (20 mg/kg, ip) for 7 days. Tramadol and Fluoxetine were dissolved in normal saline

Tail suspension test: This animal model for testing antidepressant activity is based on the principle that suspending mice suspended upside down leads to a characteristic behavior of immobility after initial momentary struggle. This behaviour reflects a state of despair which can be reduced by several agents which are therapeutically effective in human depression. On day 7 of treatment tail suspension test was conducted after 40 min of drug administration.[10] Mice were suspended on the edge of a table 50 cm above the floor by the adhesive tape placed approximately 1 cm from the tip of the tail. Immobility time was recorded during a 6 min period.[11] Animal was considered to be immobile when it did not show any movement of body and hanged passively. The total duration of immobility was recorded during the next 4 min of total 6 min test.[12] Duration of immobility was compared with control and fluoxetine groups. All results are expressed as median (range). Results were analysed using Mann Whitney test. *P*< 0.05 was considered significant.

## Results

Mean duration of immobility was significantly reduced by fluoxetine as compared to the control (*P*< 0.001). Similarly, the duration of immobility observed in mice pretreated with all the doses of tramadol was also reduced [Table 1]. Decrease in immobility due to tramadol (20 and 40mg/kg) was not found to be significant when compared to fluoxetine group.

**Table 1:** Effect tramadol and fluoxetine on immobility period in tail suspension test in mice

*Groups*	*Treatment (dose in mg/kg)*	*Median immobility period in sec (range)*
1	Control (normal saline)	121.0 (104,170)
2	Tramadol (10)	97 (56,140)*^a^
3	Tramadol (20)	84 (47,110)*
4	Tramadol (40)	52.50 (10,131)*
5	Fluoxetine (20)	56 (28,127)*

* *P*< 0.05 as compared to control

a *P*<0.05 as compared to fluoxetine (n=8)

## Discussion

In this study, antidepressant effect of tramadol was evaluated in the tail suspension test, a standard animal model predictive of antidepressant activity. Tramadol produced significant antidepressant effect at all 3 doses. The antidepressant effect of tramadol at doses of 20 and 40 mg/kg was comparable with that of fluoxetine. Similar findings were observed in an earlier study.[1] In our study, we found significantly less antidepressant activity of tramadol at a dose of 10 mg/kg as compared to fluoxetine but it was still significant as compared to the control animals.

Antidepressants (selective serotonin reuptake inhibitors; venlafaxine) by virtue of their property of mood elevation due to increase in serotonin levels also cause inhibition of release of transmitters carrying the pain sensation from nerve endings and are efficacious in chronic pain as an adjunctive treatment.[1,5,13] Similarly, it could be inferred from our study that tramadol by acting through a similar mechanism (inhibition of reuptake of monoamines leading to spinal inhibition of pain) might add a component of mood elevation to its analgesic effect. However, this needs to be verified in different models of depression.
